# Molecular Barcoding: A Tool to Guarantee Correct Seafood Labelling and Quality and Preserve the Conservation of Endangered Species

**DOI:** 10.3390/foods12122420

**Published:** 2023-06-20

**Authors:** Laura Filonzi, Alessia Ardenghi, Pietro Maria Rontani, Andrea Voccia, Claudio Ferrari, Riccardo Papa, Nicolò Bellin, Francesco Nonnis Marzano

**Affiliations:** 1 Department of Chemistry, Life Sciences and Environmental Sustainability, University of Parma, 43124 Parma, Italy; laura.filonzi@unipr.it (L.F.); pietromaria.rontani@unipr.it (P.M.R.); voccia.andrea@gmail.com (A.V.); claudio.ferrari1@unipr.it (C.F.); francesco.nonnismarzano@unipr.it (F.N.M.); 2 Department Biology, University of Puerto Rico, Rio Piedras, San Juan 00925, Puerto Rico; rpapa.lab@gmail.com (R.P.); nicolo.bellin@unipr.it (N.B.)

**Keywords:** molecular barcoding, fraud, mislabelling, species identification

## Abstract

The recent increase in international fish trade leads to the need for improving the traceability of fishery products. In relation to this, consistent monitoring of the production chain focusing on technological developments, handling, processing and distribution via global networks is necessary. Molecular barcoding has therefore been suggested as the gold standard in seafood species traceability and labelling. This review describes the DNA barcoding methodology for preventing food fraud and adulteration in fish. In particular, attention has been focused on the application of molecular techniques to determine the identity and authenticity of fish products, to discriminate the presence of different species in processed seafood and to characterize raw materials undergoing food industry processes. In this regard, we herein present a large number of studies performed in different countries, showing the most reliable DNA barcodes for species identification based on both mitochondrial (*COI*, *cytb*, *16S* rDNA and *12S* rDNA) and nuclear genes. Results are discussed considering the advantages and disadvantages of the different techniques in relation to different scientific issues. Special regard has been dedicated to a dual approach referring to both the consumer’s health and the conservation of threatened species, with a special focus on the feasibility of the different genetic and genomic approaches in relation to both scientific objectives and permissible costs to obtain reliable traceability.

## 1. Seafood Commerce and Fraud

The global fish production industry plays a crucial role in national economies, supporting an estimated 59.5 million jobs in the primary sector of capture fisheries and aquaculture [1]. Dealing with the most valuable traded food commodity worldwide, seafood has also become a fundamental income product for developing countries with net exports valued more than sugar, tobacco, meat and rice combined [1,2]. Staggering numbers highlight a constant worldwide increase both in the sector of natural seafood capture and in aquaculture production. According to available data, global fish production has reached almost 300 million tonnes [3], also considering world aquaculture, which accounts for about one third of total fish production [1]. In 2018, aquaculture fish production was dominated by finfish (54.3 million tonnes—47 million tonnes from inland aquaculture and 7.3 million tonnes from marine and coastal aquaculture); molluscs, mainly bivalves (17.7 million tonnes) and crustaceans (9.4 million tonnes) [1].

**Figure 1:**
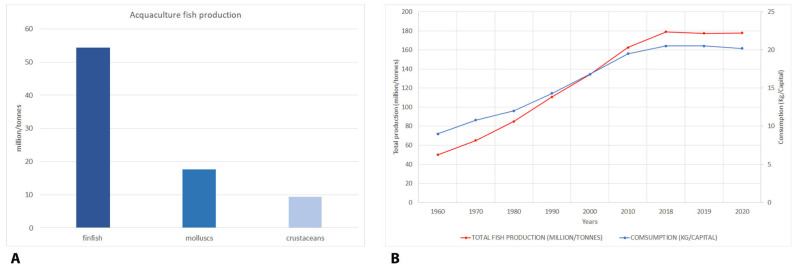
Aquaculture seafood production (million tonnes) in 2018 distributed among main production sectors (**A**); total fish production and consumption in the period 1961–2020 (**B**).

However, if there is production, there is also consumption. In fact, according to the FAO [1], global food fish exploitation increased at an average annual rate of 3.1% from 1961 to 2017, a rate almost twice than the annual world population growth (1.6%) for the same period, and higher than all the other animal protein foods (meat, dairy, milk, etc.), which increased by 2.1% per year. At the individual level, global fish consumption rose by 122% from 1990 to 2018 [1]. The annual per capita seafood consumption of fisheries and aquaculture products approximately doubled in 2018 compared to the level in the 1960s. In particular, per capita fish consumption grew significantly from 9.0 kg in 1961 to 20.5 kg in 2018, by about 1.5% per year [1]. Europeans consume, on average, 24.4 kg per person of fishery products annually, 4 kg more than the world average [4]. Despite persistent differences in levels of fish consumption between regions and states, significant trends and trajectories were observed [5]. All the above cited data are graphically illustrated in Figure 1.

The rise of international fish trade leads to the need to improve the traceability of fishery products. In relation to this, innovation must be introduced at technological level to support the consistent production increase, with a special focus on the monitoring chain assessing product handling, processing and distribution via global networks [6]. A wide number of fish species are nowadays commercialized for human consumption on a world scale, most of which derive from aquaculture production and fishery activities [7]. On the other hand, cultural improvements and attention from the media has led consumers to demand more comprehensive and precise information on fish labelling. Therefore, issues concerning food quality and safety have recently become crucial points, also considering the still-frequent habit of fish species substitutions under certain conditions.

In fact, species substitution happens to expand profits; higher value species are replaced with other less precious, often cheaper, less well-known or even illegal and protected species [8,9]. Fish traceability is nowadays fundamental to avoid substitutions that may carry hidden risks for consumers; basic consequences may be health problems that occur primarily through the consumption of cryptic species coming from contaminated areas without any sanitary checks or able to trigger allergy problems [10,11]. It must be remarked that species substitution might even occur accidentally when taxa are difficult to recognize at a morphological level, and consequently the systematics of closely related species are trivial [4].

The European Union (EU), within the renewal plan of the Common Fisheries Policy and the Common Market Organization, has introduced new requirements for the labelling of fisheries and aquaculture products through the Cape IV of Regulation EU No. 1379/2013. Although this regulation requests appropriate species traceability and labelling (scientific binomial nomenclature based on genus and species collectively with the common name), the identification of processed species is frequently difficult to perform. Morphology-based identification methods may lead to incorrect species identification. Nowadays, more innovative methods and technologies are used to assess taxa determination and authenticity. Molecular diagnostic techniques have been developed to identify food fraud using different approaches, from the use of single proteins through enzyme-linked immunosorbent assay (ELISA) [12] or species-specific DNA sequences (DNA Barcode) [13] to near infrared spectroscopy [14] or modern genomic approaches [15].

In relation to the above cited issues, the aim of this review is to highlight the main barcoding approaches to identify the most reliable ones, which are able to allow affordable taxonomic identification of cryptic seafood species and limit commercial fraud that might threaten the consumer’s health or the survival of endangered species.

The review has been prepared searching mainly in “ResearchGate”, “Google Scholar”, “PubMed”, “Scopus” and “Web of Science”. Considering that a high-quality search cannot rely on a single database, inclusion of different datasets is helpful to obtain more reliable literature. High-impact journals were preferred to avoid scarce quality of data and, above all, to have a wider audience.

## 2. DNA Barcoding and Seafood Mislabelling

In recent years, molecular barcoding (DNA barcoding) has been suggested as the gold standard in forensic taxonomy [16] and can be considered a further development of the previously applied technique proposed by Bartlett and Davidson [17]. About 30 years ago, these scientists presented an innovative methodology directed at cryptic food identification based on the use of short nucleotide regions for species authentication called Forensically Informative Nucleotide Sequence (FINS). This technique consists of a specific segment of DNA amplified using PCR and combines DNA sequencing and phylogenetic analysis to identify the most closely related taxon. Despite that, the modern concept of DNA barcoding was not established yet; the actual idea of DNA barcoding was developed in 2003 by Hebert et al. [18]. DNA barcoding is the analysis of variability in a specific genomic region, which is therefore designated the “DNA barcode”, to be compared with specific databases of previously analysed sequences that become a priori reference DNA fragments determined for the species of interest [3]. The predominant precept driving DNA barcoding is the amplification of homologous genes by means of PCR and subsequent DNA sequencing. Sequences of interest are used as a “barcode” to determine the identity and authenticity of food products, for example, for DNA identification of various plant and animal species, to discriminate the presence of different taxa in processed food and to assess the presence of raw materials in food industry processes [19]. DNA barcodes consist of a standardized short sequence of DNA (in the range 400–800 bp) that, in theory, should be easily generated and characterized for all species [20]. The central notion of DNA barcoding asserts that a short sequence of DNA should display low variability within species and greater differentiation between species [20]. Kress and Erickson [21] proposed three criteria that have to be satisfied to consider a gene region as a DNA barcode: (i) contain significant species-level genetic variability and divergence, (ii) possess conserved flanking sites for developing universal PCR primers for wide taxonomic application and (iii) have a short sequence length to facilitate current capabilities of DNA extraction and amplification. 

Nowadays, although both mitochondrial (mtDNA) and nuclear (nDNA) genes are involved in variegated approaches, the most reliable barcodes for the discrimination of different animal species are obtained using the mitochondrial gene coding for *cytochrome c oxidase 1* (*COI*) and *cytochrome b* (*cytb*) [18,22,23,24,25]. In particular, the most-used DNA barcode for seafood identification is a ~650 bp fragment of the mitochondrial gene *COI*. Many studies have shown the applicability of *COI* barcoding for accurate identification of a wide range of fish species and mislabelling detection [24,26,27,28,29,30]. 

It must be remarked that the use of mitochondrial markers for species’ correct taxonomy displays several advantages with respect to nDNA (Table 1). In particular, mtDNA has a matrilinear inheritance and is not subjected to recombination. For this reason, nucleotide variation within the same taxon is at a minimum level while, in nDNA, great differentiation emerges among different species [3]. In fact, in spite of the high mutation rate of some mitochondrial regions, *COI* and *cytb* are conserved genes within each species. 

Another advantage can be referred to the high number of copies of mtDNA contained in the same specimen that allows a more reliable amplification in case of degraded samples such as those derived from processed seafood. It is noteworthy that DNA redundancy is generated by several contemporary copies of mtDNA within the same tissue; attention must be dedicated to the correct evaluation of data, and alternative analyses using nDNA might also be considered. 

## 3. DNA Mini-Barcoding for Processed Seafood

Some of the processing and preservation techniques used with commercial seafood products might cause a limitation when full-length barcode regions (~650 bp) need to be analysed. In relation to the type of processing, the DNA may be degraded into fragments with sizes lower than 350 bp [31]. This is often the case with commercial fish, for which the correct preservation based on stable refrigerated conditions is a major issue along the entire production chain: fishing, transportation and distribution [32]. In addition, the quantity and quality of extracted DNA from processed seafood is affected by several additives, preservatives and flavours that these products contain [33]. In relation to this, a mini-barcoding approach has been suggested as valuable alternative to solve these problems and to obtain a reliable amplification of degraded DNA extracted from processed seafood. Precisely, the DNA mini-barcoding extrapolates reliable shorter DNA fragments (e.g., 100–200 bp) within the lost full length. DNA mini-barcoding is therefore a powerful method to recover DNA sequence information from specimens containing degraded DNA [23,32,33,34,35,36,37,38]. 

In particular, Shokralla et al. [33] developed an appropriate set of six mini-barcode primer pairs targeting short (127–314 bp) fragments of the cytochrome c oxidase I region. Results were successful after examining over 8000 DNA barcodes from species listed in the U.S. Food and Drug Administration (FDA) Seafood List. Authors obtained the greatest mini-barcoding success rate with the individual primer pair SH-E (226 bp). The success rate of targeting short primers reached a value of 88.6%, and it was higher than full-length DNA barcode primers that displayed a success rate of 20.5% [33]. In relation to this, Armani et al. [35] demonstrated that compared to full length *COI* barcode (655 bp), a mini barcode of 190 bp increased the success rate of PCR amplification from degraded DNA samples. In fact, 655 bp amplicon amplified from 91% (fresh) to 50% (cooked) and 81% (ethanol-preserved) samples, while the proposed 190 bp amplicon amplified 100% (cooked) and 94% (ethanol-preserved) samples. Chakraborty et al. [39] found that a 154-bp fragment from the transversion-rich domain of 1367 *COI* barcode sequences can successfully delimit species in the three most diverse orders of freshwater fishes (Cypriniformes, Siluriformes and Perciformes). Interestingly, variegated approaches can be applied according to the researcher experience and the type of product: Sultana et al. [38] proposed a novel mini barcode marker (295 bp) to discriminate fish species in both raw and processed states, while Pollack et al. [40] analysed in the same year the effects of cooking methods on DNA integrity using both full-length (655 bp) and mini-barcodes (208–226 bp). The highest overall success rate was found for one of the tested mini-barcodes (SH-E mini-barcode), regarding canned samples. Recently, Filonzi et al. [32] performed molecular analysis of 71 commercial fish samples based on mini-*COI* sequencing, testing two different primer sets. The first pair of universal primers called “Fish_mini” (295 bp) was the one proposed by Sultana et al. [38], while the second primer pair (SH-E, 226 bp) called “Fish_miniE” was tested following the protocol suggested by Shokralla et al. [33]. “Fish_miniE” successfully amplified 62 samples out of 71, reaching an amplification success rate of 87.3%, while “Fish_mini” displayed a positive result in 69 of 71 samples and a success rate of 97.2%.

It is noteworthy that the DNA mini-barcoding method might show loss of discriminatory power, especially for species that are closely related. Therefore, a key point to select a DNA barcode region is the presence of enough polymorphisms to allow discrimination among species in combination with high homology to design common primers [3]. From this point of view, the *COI* sequence of a small mini-barcode fragment (≥100 bp) carries the information required for identification of individual species with more than 90% of resolution [33]. However, it must be remarked that when the barcode length is too short (≤150 bp), further problems may emerge. In fact, despite the fact that the DNA region could be easily amplified, the correct species attribution might not be retrieved via direct comparison, and an erroneous identification could happen due to overlapping of closely related taxa [38].

## 4. The Impact of Seafood Frauds on Human Health

As mentioned above, a food fraud is committed when food is illegally placed on the market, most of the time for financial gain. Deliberate mislabelling and replacement of high-value species with cheaper ones is defined an Economically Motivated Adulteration (EMA) and is considered fraud [41]. In a specific document published in 2013 by the European Parliament, seafood was identified as the second most likely food item to be subject to fraud, following olive oil [42]. This voluntary or involuntary practice can also lead to unexpected events concerning different aspects of human health (from food allergies to poisoning and to multiorgan disfunctions). For example, roasted fillet products of codfish called Xue Yu (order Gadiformes) are largely consumed in China [43]. According to Xiong et al. [44], out of a total of 153 samples of roasted Xue Yu fillet products collected from 16 cities in China, only 42% of the samples were identified as belonging to Gadiformes, while the others were Scorpaeniformes, Tetraodontiformes and Lophiiformes. Moreover, the identification of poisonous *Lagocephalus* spp. from 37 samples highlighted the danger of mislabelling for human health. The consumption of this poisonous fish leads to several diseases: diarrhoea, body and organ (liver, kidney) weight loss, oxidative stress evidenced by an increase in lipid peroxidation (TBARS) and, conversely, a decrease in activities of such antioxidant enzymes as SOD, catalase and GSH-Px in different tissues (blood cells, liver, kidneys) as well as a decrease in alanine aminotransferase (ALT) and alkaline phosphatase (ALP) concentrations detected in blood plasma [45]. Furthermore, some species’ flesh can be toxic if not treated properly. Lowenstein et al. [46] showed how several New York City sushi restaurants sold Escolar (*Lepidocybum flavorunneum*) as a variant of ‘‘white tuna”. Escolar is a species banned for sale in Italian and Japanese markets due to high-risk health concerns. The large amount of wax esters in the lipidic fraction of its raw flesh is responsible for toxic activity over the gastroenteric apparatus [47]. Some species such as *Solea solea*, *Pleuronectes platessa* and *Merluccius merluccius* were mislabelled with less valuable species including *Pangasius hypophthalmus* in South Italy [48]. Pangasius farms were found contaminated by heavy metals in the Mekong River (Vietnam) with a potentially unhealthy status for human consumption [49]. 

## 5. Implications for the Conservation of Endangered Species

Another important aspect is that food fraud threatens the conservation of several endangered species. The International Union for the Conservation of Nature (IUCN) was founded in 1948 and was the first international organization to deal with environmental sustainability. It soon became the world’s most comprehensive association dedicated to the global conservation of endangered animal, fungi and plant species. Among IUCN tools, the Red List is a fundamental indicator of the conservation status of the world’s biodiversity. Far more than a list of species, it is a strategic document to inform and define guidelines for species preservation, helpful for policy makers both at regional and global levels [50]. In particular, the “The IUCN Red List of Threatened Species” reports all living beings showing conservation issues, listed in different categories of severity: NE (not evaluated), DD (data deficient), LC (least concern), NT (near threatened), VU (vulnerable), EN (endangered), CR (critically endangered), EW (extinct in the wild), RE (regionally extinct), EX (globally extinct) [50].

One of the most common seafood frauds based on a species substitution occurs in the swordfish (*Xiphias gladius*, NT) trade [51]. *X. gladius* species substitution usually involves the taxon Selachimorpha. Currently, 153 shark species are classified as vulnerable (VU), endangered (EN) or critically endangered (CE) by the International Union for the Conservation of Nature [50]. Furthermore, 14 of them are listed in Appendix I, II or III by the Convention on International Trade in Endangered Species of Wild Fauna and Flora (CITES), which regulates their international commercial trade. The fact that the flesh of many fish species is similar in appearance, taste and texture [1] means that fraudulent practices could be unnoticed, especially in processed fish products. Ferrito et al. [51] reported the identification of the species *Prionace glauca* (Blue shark, NT), *Mustelus mustelus* (common smoothhound, EN) and *Oxynotus centrina* (angular roughshark, EN) in slices labelled as swordfish bought in fishmarkets in Southern Italy. Shehata et al. [52] reported a case of an individual of *Sphyrna lewini* (scalloped hammerhead, classified EN) mislabelled as *Prionace glauca* (blue shark, NT). A similar finding was previously reported by Willette et al. [53]. This fraudulent mislabelling of fish products is quite widespread and affects other endangered species. Juveniles of *Thunnus obesus* (VU), *T. alalunga* (LC), *T. albacares* (LC) and *T. thynnus* (LC) are sold as anchovy [54]. Although some of these species are considered LC on a global scale, most of their populations are seriously threatened at the regional level. This is particularly the case of the Mediterranean Sea. Anyway, Selachimorpha is one of the most mislabelled taxa. The shortfin mako *Isurus oxyrinchus,* which has recently been assessed in the “EN” category [50], is often sold, similarly to the Lamnidae, *Lamna nasus* (VU) (Porbeagle), as swordfish in some fish markets of Santiago (Chile); respectively, 2.13% of swordfish samples were shortfin mako and 6.39% were identified as porbeagle [55]. Interestingly, French and Wainwright [56] used DNA barcoding to identify the presence of shark DNA in pet food commercialized in Singapore. The most common identified sharks were the blue shark *Prionace glauca*, an overexploited species, and *Carcharinus falciformis* (silky shark), the latter listed in CITES Appendix II and “vulnerable” in the Red List [50]. In Filonzi et al. [32], a barcoding investigation was carried out in Italian fish markets over the last 10 years. Results have shown an improved situation compared to previously presented data from the same authors, witnessing a general improvement in the management and control of Italian fish markets [24]. Nevertheless, two samples labelled as *Katsowomus pelamis*, not directly involved in conservation issues, turned out to be either *Thunnus thynnus* (LC) or *Thunnus maccoyii* (EN). Similarly, *Lamna nasus* (VU) was *Isurus oxyrinchus* (EN), a more endangered species [32]. According to Lowenstein et al. [46], 19 out of 68 sushi products based on tuna purchased from 31 restaurants in Manhattan (New York City) and Denver (Colorado) were *T. thynnus* (LC) or the endangered southern bluefin tuna (*T. maccoyii*, EN), though 9 out of 31 restaurants that were involved did not list these species on their menus.

Interestingly, two bluefin tuna species, yellowfin and albacore, are no longer critically endangered, and the recent revision of IUCN classification has removed these species from the major risk categories [50]. The unexpectedly fast recovery bears witness to the effort dedicated over the past decade to end overfishing and limit unauthorized trading. On the other hand, researchers caution that, unlike tunas, many other marine species remain highly endangered. In fact, more than a third of the world’s sharks and rays are still threatened to extinction due to overfishing, illegal commerce, habitat loss and climate change. 

A further problem related to conservation of endangered species concerns the habit of using same/similar common names generating a sort of confusion over different taxa. For example, in the internal market of Brazil, which is an important country managing shark fisheries, besides being one of the largest importers of shark meat worldwide, several elasmobranch species are traded under the common popular name “caçao”. In 2018, Almeròn Souza et al. [57] published a study about “caçao” fish, analysing 63 samples with DNA barcoding based on mitochondrial *COI* gene. As a result, they found DNA coming from 20 different species, 18 of which belonged to seven elasmobranch orders. It is noteworthy that some species belonged to such evolutionary distant taxa as *Xiphias gladius*. Considering IUCN criteria, 47% of the detected elasmobranch species were threatened at the global level, while 53% were threatened and 47% critically endangered in Brazil [57].

As shown in Table 2, seafood fraud is a common widespread practice all around the world. Sharks and unidentified local fish are the most involved species. In some cases, mislabelling also involves endangered species, frustrating fauna conservation measures. It must therefore be remarked that DNA barcoding is one of the most effective analysis tools to discover dangerous, unhealthy and unethical activities in commercial fisheries. The capability of mitochondrial and nuclear markers to allow fast identification processes has represented a turning point not only for food safety issues but also in the field of wildlife crime investigations. Interestingly, molecular tools not only support a fast taxonomic identification but may also define population genetic parameters. 

## 6. DNA Barcode Regions

### 6.1. Cytochrome c Oxidase I (COI) Gene

The *cytochrome c oxidase I* (*COI*) gene is the preferred sequence that serves as a “barcode” to identify and delineate the animal life form [22]. This DNA barcoding region is called the Folmer region and consists of a 648–655 bp long DNA segment near the 5’ end of mitochondrial cytochrome c oxidase subunit 1. This region was first proposed by Folmer et al. [60] to identify metazoans and used in several studies on insects [38,61], mammals [62,63], amphibians [64], reptiles [65] and birds [18]. Considering birds, Hebert et al. [18] described a success rate of species identification from 98% to 100%. Ward et al. [26] proposed the *COI* mitochondrial gene as a barcode marker in fish. In particular, 207 species, mainly Australian marine fish, were sequenced for the 655 bp region of the mitochondrial cytochrome oxidase subunit I gene, demonstrating that cryptic fish species are revealed through the discovery of deep divergence of *COI* sequences within currently recognized species. However, to recover the variable segment flanking on both sides of the *COI* region, multiple rounds of PCR and multiple primer combinations were required [26]. Ivanova et al. [27] improved the *COI* barcoding protocol through identifying a primer set that could ensure wider use on more taxonomic groups, focusing on fishes. Primer cocktails were tested in 94 fish to identify the different *COI* sequences. Interestingly, the COI-2 primer cocktail was developed for mammalian barcode region amplification [63] but was proven to work in fish as well. Conversely, the COI-3 cocktail developed for fish was very effective in mammals, amphibians and reptiles. Together, these cocktails amplified the barcode region for every tested species [27]. The mitochondrial *COI* gene has been implemented as the preferred barcode region for the animal kingdom because it provides regular resolution at the species level and it is a region present in a large number of species [34,66]. For this reason, in the following years, *COI* barcoding became a standard method for the identification of fish specimens and products, Ref. [59] due to the need of finding adequate tools to confirm species authenticity and product labelling to avoid commercial fraud [59,67,68]. Seafood authentication and food safety became a wide scientific interest all over the world. In this regard, Filonzi et al. [24] developed molecular barcoding using 650 bp of *COI* gene to identify seafood frauds in Italy. A special focus was put on Italian commercial markets during 2008 when the results were obtained in 69 processed fish products belonging to 27 teleost species. DNA barcoding using Sanger’s sequencing revealed incorrect labelling in 22 samples (32%). Among the replaced species, 18 (26%) were severe frauds from both economic and nutritional perspectives [24]. Armani et al. [35] dedicated further attention to ethnic seafood sold in the Italian market. Sixty-eight variously processed ethnic seafood products were collected from the Italian market and full cytochrome c oxidase DNA barcode (FDB, ~655 bp) or a mini-*COI* barcode (MDB, ~139 bp) were performed. Discrepancies between labelling and molecular identification were revealed in 48.5% of all products. In particular, two samples were labelled as squid but identified as *Lagocephalus* spp., which is a poisonous puffer fish species banned from the EU market. This result confirmed the importance of DNA barcoding as a golden tool for protecting consumers’ health and economic interests [35].

During recent decades, using the analysis of the *cytochrome c-oxidase I* gene sequence, seafood mislabelling was identified in different countries of Europe such as Germany [37], Italy [32,69], United Kingdom [8,70], France [71], Spain [72], Greece [73], Portugal [74] and Northern Europe [75]. The work proposed by Tinacci et al. [76] underlined the urgency to review and update the Bulgarian official seafood list. Ninety-seven labelled seafood products collected from Bulgarian wholesalers were analysed using COI barcoding and revealed a species substitution rate of 17.7%. The analysis of the official seafood denomination label highlighted the presence of commercial and scientific names not included within the official list (59.2%), the lack of a scientific name (34.1%), the incomplete reference to the catching area (85.2%) and the absence of the fishing gear (55.2%). Pardo et al. [77] focused their studies on fish mislabelling rate in the mass caterer (HoReCa) sector across Europe. A total of 283 samples were collected in 180 mass cafeterias inside commercial outlets in 23 European countries. Molecular analysis based on the *COI* gene sequence revealed that 26% of the samples were mislabelled.

Several studies were performed in different places such as South America [28,78], Asia [79,80,81], the US [53,82,83], Africa [84,85] and Australia [86,87]. In relation to this, Cawthorn et al. [88] estimated the prevalence of species substitution and fraud prevailing in commercial fish in the South African market. The region of the *COI* gene was sequenced from 248 fish samples collected in seafood wholesalers and retail outlets. DNA barcoding was able to provide unambiguous species-level identifications, and 9% of samples from wholesalers and 31% from retailers were identified as different species from the ones indicated. Munguia-Vega et al. [89] conducted a DNA barcoding study in three cities within Mexico and sequenced the *COI* gene in 376 fish samples sold as 48 distinct commercial names at fish markets, grocery stores and restaurants. Overall, the study mislabelling rate was 30.8%. Dissimilar mislabelling rates [3,90] have been shown depending on countries, species groups, the dealer, strict regulation of government, the processing of fish and the year. In Europe, for example, Bénard-Capelle et al. [71] detected only 14 mislabelling cases (3.7%) among the 371 samples collected in France. On the contrary, the average seafood mislabelling in Belgium was 31.1%, while the substitution of bluefin tuna was up to 95% [75]. A similar scenario characterizes the rest of the world; indeed, high rates of mislabelling, using the *COI* gene, were observed in North America, respectively, 47% in the USA [53] and 41.2% in Canada [91]. In contrast, in South Korea, one of the biggest seafood markets in the world with strict laws of the Korean Government, a low mislabelling rate was found by Do et al. [92]. This study used the *COI* gene to investigate mislabelling of 157 seafood samples; 12 mislabelling cases were found with a mislabelling rate of 7.6%. A wide variety of fish products have been studied using DNA barcoding, but little investigation of sushi, poke and ceviche dishes sold at restaurants has been performed. In relation to this, Kitch and colleagues [93] dedicated their research to this type of product based on raw fish, analysing 105 samples collected in Orange County (CA). Among these, 103 samples were positively identified using DNA barcoding and a species substitution rate of 23.3% was evidenced. On the other hand, non-congruent labelling was found in 45.6% of samples, while 63.1% of samples had some form of mislabelling. When the assessment was stratified in relation to the product category, ceviche had the highest overall mislabelling rate with 85.3%, followed by poke with 61.8% and sushi with 42.9%. Some authors [72,93] hypothesized that a general lower rate occurred in specimens obtained from local grocery stores in comparison with larger supermarkets, wholesalers or restaurants.

At last, the suitability of the *COI* gene for species identification using the mini-barcode version must be remarked. The proposal of a wide set of primers [33] allows the application of this approach to detect short informative fragments useful for analysing particularly degraded samples (see dedicated Section 3 in this review). 

### 6.2. Cytochrome b (cytb) Gene

In 1989, Kocher et al. [94] identified the cytochrome b region as a possible genetic marker for species identification. A standard set of primers headed for conserved mtDNA regions was amplified and sequenced in more than 100 animal species, among which were mammals, amphibians, birds, some invertebrates and fishes. The results of this study showed different genetic variability between different species of the same biological class. This unexpected taxonomic utility of cytochrome b (*cytb*) primers provided opportunities for phylogenetic and population research [94]. Several studies proposed mtDNA for species identification, considering this particular genome one of the most useful for phylogenetic work [95,96,97,98]. At the beginning of the twenty-first century, the mitochondrial *cytb* gene was probably the best-known mitochondrial gene in terms of the structure and function of its protein product [99,100]. Parson et al. [101] attempted to identify DNA from 44 different animal species covering the five major vertebrate groups (15 mammals, 22 birds, 1 amphibian, 2 reptiles, and 4 fish species). The *cytb* fragment was amplified, sequenced and compared to the database’s homologous 300 bp fragment sequence. Similarly to Ivanova et al. [27], who defined the best primer set to assess the correct taxonomic groups using *COI* sequences (see previous chapter), Sevilla et al. [102] tried to improve the *cytb* barcoding protocol for fish. A set of 21 PCR primers and amplification conditions were developed to barcode any teleost fish species according to their mitochondrial cytochrome b gene sequences. Overall, the above procedure yielded > 99.9% successful amplifications [102]. In fish, *cytb* has also proved to be a useful marker for the identification of seafood species and/or resolving species phylogenies [25,103,104,105,106]. In particular, mt *cytb* has been used to identify flatfish, gadoids, anchovies, eels, scombroids and many other species [107,108,109,110,111,112]. *Cytb* barcoding as a molecular method involving DNA sequencing can be successfully used for fish species identification, giving a key contribution to the correct labeling of fish products [6,24,113,114]. For instance, using *cytb* sequences, Marko et al. [6] showed that 70% of 22 red snapper samples (*Lutjanus campechanus*) from US markets were less-valuable species of Lutjanidae. 

Armani et al. [115] analysed the genera *Neosalanx* and *Protosalanx* belonging to the *Salangidae* fish family, also known as icefish or silverfish, which is imported processed from China to Italy. Direct gene sequencing was carried out to taxonomically classify the correct species in 10 specimens of *Neosalanx taihuensis* directly collected from Lake Taihu. In addition, 200 specimens of icefish whose indirect origin was attributed to 40 markets (27 from Italy and 13 from China) were analysed with the same technique. The main purpose of the taxonomic approach was to investigate any potential mislabelling. Obtained *cytb* sequences identified 90% of market samples as *N. taihuensis*. The data rose to 93% when only products collected in Italy were considered. Interestingly, 15% of samples coming from Italian markets were mislabelled, thus confirming the existence of commercial fraud at an international level. In similar research, Ha et al. [116] carried out an analysis of 10 processed fillets collected from department stores in Hanoi (Vietnam). Only four of these products matched common and corresponding scientific names after the appropriate barcoding evaluation. The other six samples exhibited inappropriate labelling, switching from *P. hypophthalmus* into *P. boucourti*. Although no real commercial fraud was found in these products, the correct scientific names of fish species should always be considered for elaborated products as they are publicly available in supermarkets where people have no specific consciousness of this problem [116]. Meanwhile, Cutarelli et al. [117] analysed 60 samples of canned fish belonging to three genera and five species collected by Italian Health authorities: PIF (Border Inspection Posts), NAS (Anti-Sophistication Police) and ASL (Local Health Authority). The species declared were confirmed in all samples except two, which were labelled as *Thunnus alalunga* instead of *Thunnus thynnus*. All other samples were correctly labelled as *Thunnus albacares*, *Thunnus obesus, Sardina pilchardus* and *Engraulis encrasicolus* [117]. Gomes et al. [118] evaluated the authenticity of 107 frozen fillets tagged as Gurijuba (*Sciades parkeri*) and Uritinga (*Sciades proops*) bought from local markets located on the northern Amazon coast. About 16% of fillets initially attributed to *S. parkeri* were substituted with *S. proops*. Forensic analysis using mtDNA markers proved to be highly efficient in the discrimination of processed seafood, providing unequivocal taxonomy. In fact, commercial fraud pertaining to Gurijuba fillets was discovered using *cytb* sequences as a barcode in fish [118]. The same markers were adopted by Souza et al. [119] to estimate the mislabelling prevalence of seafood displayed in street markets, fishmongers, supermarkets and restaurants of Rio de Janeiro (Brazil). Analyses were carried out between 2012 (*n* = 77) and 2020 (*n* = 183). Nearly 50% (130/260) of the analysed products had no correspondence. It is noteworthy that the frequency of mislabelling varied across the commercialization chain. Once again, a split evaluation displayed higher mislabelling values detected in street markets (61%) and restaurants (82%) compared to fishmongers (38%) and supermarkets (22%). The most commonly exploited species as substitutes were *Pangasianodon hypophthalmus* (75%) and *Xystreurys rasile* (17%). Substitute taxa were usually lower priced, supporting an economic motivation as the general idea formulated for mislabelling. These results reinforce the need for updated and consistent regulations addressed to a more stringent control of sales of a wide variety of species in street markets and restaurants [119].

### 6.3. 16S rDNA and 12S rDNA

The two ribosomal RNAs, *12S* rRNA (819 to 975 bp in vertebrates) and *16S* rRNA (1571 to 1640 bp in vertebrates) genes, similarly to other mitochondrial genes, have numerous nucleotide substitutions, suggesting their use as a tool for species identification [120]. In relation to this, the mitochondrial *12S* rRNA and/or *16S* rRNA genes have been used as molecular markers to identify mammals, birds, shrimp and other species [121,122,123,124,125,126,127].

In particular, the mitochondrial gene coding for *12S* rRNA has been reported to be an excellent tool for the authentication of fish and seafood due to its mutation rate, acceptable length and availability of sequence information in databases [128]. Meanwhile, there is evidence that the *16S* rDNA is adequate for discriminating some *Epinephelus* and *Mycteroperca* species from non-target species [129]. Various studies were carried out to investigate and implement the use of these rRNA genes as molecular markers for species identification. Variants belonging to *12S* and *16S* molecular markers have been used to identify a wide variety of flatfish, eel, cardinalfish, cephalopods, mackerel, hairtail species, crab and several others [39,128,130,131]. Worldwide, *12S* and *16S* rDNAs have become valuable barcoding tools to assess seafood fraud verification cases. Their application to high-value fish has been demonstrated by Von der Heyden et al. [132], who tested several widely available and generally expensive fish in South African high-priced markets utilizing mtDNA *16S* rRNA sequencing. Interestingly, half of 178 tested samples revealed mislabelling with a special focus on kob. In fact, 84% of kob fillets, *Argyrosomus* spp, provided an attribution to other species, such as mackerel, croaker and warehou. Phylogenetic analyses supported the general idea of frequent species substitution among barracuda, wahoo and king mackerel. Genetic analyses gave the opportunity to reveal that red snapper fillets included those of river snapper *Lutjanus argentimaculatus*, which is a prohibited species for sale in South African markets. From preliminary population genetic comparisons, some 30% of kingklip samples probably had their origin in New Zealand rather than southern Africa. A similar mislabelling rate was found in seafood products sold in South America. In northern Brazil, an investigation was carried out for critically endangered species’ (*Pristis perotteti,* largetooth sawfish) commercialization in fish markets, typically labelled as “sharks”. Based on partial DNA sequences of the mitochondrial *16S*, 55% of samples were unequivocally identified as *P. perotteti*, while the others (45%) belonged to the families Carcharhinidae and Ginglymostomatidae [133]. Considering one of most exploited species in Caribbean regions, also including part of South America, Lee-Charris et al. [134] focused their attention on the common snook *Centropomus undecimalis*, realizing that populations drastically decreased in that marine sector due to overfishing and environmental degradation. Thus, there is a market imbalance between the availability of snook products and their demand by consumers, which generates such fraudulent actions as species substitution. Therefore, to investigate the existence of mislabelling in common snook products, 15 fresh snook fillets from six of the main fish markets and 44 frozen snook fillets from the five commercial brands available in Santa Marta (Colombia) were identified through molecular barcodes (*16S* rDNA and *COI*). A sort of discrepancy emerged between department stores and local shops. In fact, an astonishing 98% of processed fillets were found to be fraudulent in the commercial markets in comparison to a much lower involvement of fish shops, where only a single case was registered. The species used to substitute snook included the Pacific bearded brotula *Brotula clarkae* (38 samples), the Nile perch *Lates niloticus* (4 samples) and the acoupa weakfish *Cynoscion acoupa* (1 sample) [134]. To contribute to the current knowledge on mislabelling rates in Europe, some studies have been performed on seafood products in Spain [135,136]. Horreo et al. [135] tested 77 fish dishes from 53 different restaurants located in nine districts of Madrid (Spain). A short fragment of the *16S* rDNA was sequenced and compared with sequences in databases to verify that seven species or genera and almost 30% of the samples were mislabelled. Mislabelling was present in 37% of the sampled restaurants and 71% of the sampled districts. 

In more recent years, several researchers have verified the presence of seafood fraud in Asia [137,138,139]. Hossain et al. [137] experimented with a pair of universal primers with the aim to target a 198 bp fragment of the mitochondrial *16S* rDNA to assess species identification in partially degraded samples. The *16S* rRNA gene was tested on 24 processed fish products commonly consumed in Malaysia. The newly developed marker successfully identified 92% of the tested commercial fish products based on 96–100% sequence similarities. Five out of 24 (20.8%) fish products revealed a considerable degree of species mislabelling. According to its limited fragment length, the new molecular marker developed in Hossain et al. [137] is a reliable tool to identify fish species even in highly processed products. Results were particularly helpful to detect species substitutions with the objective to protect both consumers’ health and economic interests [137]. Xing et al. [138] compared the results obtained using the shorter sequence of the mitochondrial region of *16S* rDNA (~220 bp) with a region of *COI* gene to test a variety of sold animal-derived food products in the Chinese market. More precisely, 52 samples, quite variegated and including meat, poultry and fish purchased from retail companies and online sources, were assessed. Approximately 94% of the samples generated barcode sequences. On the opposite, the failure rate for barcodes based on the entire *COI* region was 44%. Despite this, they obtained valuable data using the *16S* rDNA mini-barcode from 87% of the *COI*-failed cases. Overall, the survey revealed that 23% of animal-derived products were mislabelled and, in most cases, contained undeclared species [138].

A higher mislabelling rate was also found in canned tuna sold in Taiwan. Chang et al. [139] sequenced two mitochondrial regions, *16S* and the control region (D-Loop), to assess 90 canned tuna products, also including 25 animal food items. Results revealed that *Sarda orientalis*, *Euthynnus affinis*, *Auxis rochei* and *Auxis thazard* are commonly exploited as substitutes in place of declared tuna products. Specifically, only 63.33% of investigated samples are true canned tuna, i.e., containing *Thunnus* species or skipjack tuna [139]. On the other hand, a low mislabelling rate was found in the study of Helgoe et al. [140], in which Atlantic cod (*Gadus morhua*) samples (*n* = 546) were collected from local markets, supermarkets and restaurants from eight cities across Spain. DNA barcoding was performed used PCR-based assays of the mitochondrial cytochrome oxidase-I (*COI*) and *16S* rRNA loci. A 6.2% mislabelling rate was discovered. Although no evidence emerged for possible distinct geographic patterns of mislabelling, biologic samples obtained from restaurants were more likely to be mislabelled than those sampled in department stores. In relation to sample preparation, such processed products as elaborated or salted/smoked fish were more likely to be mislabelled than fresh or frozen products. Common ling (*Molva molva*), haddock (*Melanogrammus aeglefinus*), saithe (*Pollachius virens*) and Alaska pollock (*Gadus chalcogrammus*) were the most common substitutes, while Nile perch (*Lates niloticus*) and striped catfish (*Pangasianodon hypophthalmus*) were the most taxonomically dissimilar to Atlantic cod.

### 6.4. Nuclear DNA

Despite the advantages of using mtDNA for species identification, some nDNA targets have also established to be successful in the recognition of fish and seafood species. Frequently, nuclear genes have been used in combination with mitochondrial genes to improve the efficiency and robustness of the analysis. In fact, to overcome limitations connected to mtDNA in defining closely related taxa, different regions of nDNA have been proposed as helpful markers to reach a reliable identification. In particular, nuclear regions are fundamental to solve issues related to hybridization and introgression [3]. The most common is the nuclear *5S* rRNA gene (*5S* rDNA), which consists of a small 120 bp conserved region coding for *5S* rRNA and a variable region of noncoding DNA, termed the non-transcribed spacer (NTS), that has a species-specific length and sequence [141,142,143]. In particular, the *5S* rRNA gene has been used to identify gadoids, salmonids, sharks, mackerel and others [143,144,145,146]. Perez and Garcia-Vazquez [147], using PCR amplification and sequencing of the nuclear *5S* rDNA, demonstrated that hake products commercialized in southern European (Spanish and Greek) market chains presented more than 30% of incorrect species labelling. DNA analysis showed that tails and fillets were more mislabelled than other products, and African species were substitute species for products labelled as American and European taxa [147]. Triantafyllidis et al. [148] used the *5S* rRNA gene as molecular marker for detecting and quantifying allergenic fish species contained in Greek commercial seafood labelled with generic and unspecific names. Almost 85% of the analysed products contained highly allergenic hake or grenadier species and only 15% contained less histaminergic species such as cod and haddock [148]. Frigerio et al. [149] performed a new set of primers on the *5S* rDNA and non-transcribed spacer (*NTS*) on 27 processed and unprocessed products collected at the Milan fish market and across different Italian supermarkets. In their research, a new DNA mini-barcoding region suitable for species identification was identified [149].

Another important nDNA region which has been used for species identification is the intronless nuclear rhodopsin gene (*RHO*) [150], which has been proposed for recognizing teleost fishes by Sevilla et al. [102]. Under certain circumstances, such as hybridization and introgression that are frequent in fish populations, the intronless nuclear rhodopsin gene results to be a useful additional marker for fish species identification in combination with mtDNA targets [91,151]. In particular, Abdullah and Rehbein [152,153] demonstrated the usefulness of the rhodopsin gene as nuclear marker for fish species differentiation, particularly tuna food products from Indonesian markets.

Variations in the gene coding for *18S* rDNAs allowed the identification of four species of abalone in Thailand, *Haliotis asinina*, *H. ovina* and *H. varia* [154]. Several authors used the *18S* rDNA for fish identification [155,156,157]. *18S* rDNA-barcode possesses several advantages: the complete *18S* rRNA gene sequence displays different variability levels [158]; a well-developed *18S* rDNA-sequence library, including a wide spectrum of taxa, is available on Genbank and facilitates the identification of variable and conserved regions; the presence of multiple nuclear copies [159] increases the probability of amplifying degraded prey DNA from predator faeces and, thus, increases the sensitivity of the method [160]. 

Finally, the myosin heavy chain 6 cardiac muscle alpha gene (*MYH6*) has been used to identify genetic diversity between species [161,162]. In particular, Ramirez et al. [161] proposed this gene to highlight the phylogenetic relationships among the *Laemolyta* genus. Twenty-four samples of four valid nominal *Laemolyta* species were investigated using barcoding analysis.

## 7. DNA Barcoding Databases

The organization and storage of sequences in several comprehensive and consistent barcode databases is one of the major strengths of the DNA barcode-based technologies related to species identification, authentication and phylogenetic analysis [163,164,165].

In most cases, each database contains an organized set of information concerning species name, voucher data, collection records, specimen identifiers and genetic sequences [164]. The computation of nucleotide variation, using evolutionary models such as the Kimura2-parameter distance method [166], allows to assess the correct match between the target and reference sequences. To ensure the correct identification, the database must contain reference sequences representing that species; this implies the accuracy of the association between the loaded sequences and the referred species, and this may be a problem especially in seafood cases due to many morphological similarities between different species and taxa [167]. 

DNA barcoding is successfully applied to seafood for many reasons: (1) in comparison to other animal sources (e.g., cattle, sheep, goat, horse) where taxonomic discrimination must be carried out at race level, the assessment of seafood is mainly performed on a high number of species and the effectiveness of this technique is therefore enhanced; (2) classical identification approaches are not useful in many cases, particularly with processed food; (3) in seafood more than in other living groups, molecular identification can go further than the species level, allowing in several cases the identification of varieties that belong to local natural resources and hence the identification of the geographical origin of a certain product [163].

The support of bioinformatics associated with the laboratory approach is therefore fundamental. One of the most popular public databases is GenBank, which is part of the NCBI (International Nucleotide Sequence Database Collaboration) that also includes the DNA DataBank of Japan (DDBJ) and the European Nucleotide Archive (ENA). Conceived in 1982, at this time, there are more than 200 million sequences (release Aug 2022: 239,915,786 sequences). From 1982 to the present, the number of bases in GenBank has approximately doubled every 18 months [168]. GenBank also has a useful tool that provides a rapid sequence comparison to identify unknown species. This software, called BLAST (Basic Local Alignment Search Tool), directly approximates alignments that optimize a measure of local similarity between sequences and calculates the statistical significance of matches [169]. This database is also widely used in food safety to perform correct specimen identification due to the many available sequences. Despite this, it has been criticized as being susceptible to problems such as incorrect species identification and missing information [170], especially with fish and seafood [171].

It must be remarked that despite the large number of sequences so far deposited in GenBank, the database is not appropriately dedicated to seafood and, therefore, a lack of information or unreliable taxonomy may complicate correct species attribution [3]. For this reason, more specific databases dedicated to barcoding or fish species are more helpful for the solution of traceability issues.

Alternatively to GenBank, the Barcode of Life Database (BOLD) is a cloud-based data storage and shared platform that supports the assembly and use of DNA barcode data. It has been created by the organization iBOL, which has the main purpose of developing a globally accessible DNA-based system for the discovery and identification of all multicellular life [172]. Currently, BOLD consists of more than 11 million sequences belonging to 243,000 animal species. All the stored barcodes have been deposited after identification by expert taxonomists. Moreover, BOLD has a useful component called BOLD-IDS, an identification engine tool that provides, whenever possible, a match between the target barcode sequence and the referred one, allowing a taxonomic assignment. 

Both BOLD and Genbank concern all species and, in food safety, they are widely used for specimen identification of many products, from raw to processed food [163], but also for the detection of parasite and pathogens in human and animal food [173,174]. BOLD identification normally achieves higher acceptability and scientific merit since it is based on verified sequences and tagged specimens. However, BOLD records suffer from some shortcomings such as a low number of species, and it usually depends on GenBank sequences. Some pitfalls were documented, such as mistakes in private submission or records gleaned from the GenBank database that can create incorrect identification when BOLD-IDS are used [38,175].

With the aim to collect and assemble standardized DNA barcode sequences within a well-organized reference library to aid the molecular identification of all fish species, the Fish Barcode of Life Initiative (FISH-BOL) is a concerted global research project launched in 2005 [164,176]. FISH-BOL currently has DNA barcode records in place for nearly 8000 species. So far, several studies have shown a high success rate of DNA barcoding for species identification [177] using FISH-BOL, with 93% of freshwater species and 98% of marine species tested with unambiguous taxa differentiation [167]. 

Fish-Trace is a public European database which has been created to compile and deliver accessible data and material needed for the genetic identification of marine fish species in Europe. Fish-Trace consists of 220 species belonging to 75 different families [178]. To minimize the risk of incorrect association between the loaded sequence and the referred species, all the fish specimens have been identified by taxonomists and stored in natural history museums [179]. Every sequence loaded contains metadata information of both sampling and geographic origin. The Fish-Trace catalogue is based on two genes, one mitochondrial (*cytb*) and one nuclear (*rhodopsin*), used in analysis validation and quality control. Although Fish-Trace is specifically dedicated to fish species, its data are limited to European species based only on two genes, not on *COI*, which nowadays represents the most reliable one. 

Further, Aquagene is a free-access database of genetic information of marine species. This database now consists of 603 species referenced by 1093 individuals and 1383 barcodes. All species are characterized by multiple gene sequences including the standard COI barcoding gene together with *cytb*, *MYH6* and *RHO*, therefore facilitating unambiguous species determination even for closely related species or those with high intraspecific diversity. Moreover, it is possible to find data concerning the sampled specimen, such as digital images, voucher number and geographic origin [180].

It is noteworthy that one of the strengths of this technology is the availability of many sequences stored in multiple databases. Although this may allow reliable comparisons, it can also generate a certain degree of complexity in species identification due to different variations on the information sources. This usually occurs more often in seafood identification due to many morphological similarities in closely related groups [167]. As shown in Table 3, expert-verified databases present a lower number of species, while NCBI Genbank, despite a large number of species and sequences, suffers from the absence of data validation and a lack of sample information [179]. In this regard, in some cases, the same barcode produces different results in those databases [32,38]. For this reason, integration of different databanks coupled to an expert based judgment is fundamental for reaching a reliable specific attribution, integrating the result provided from the databases and the sampling information [32].

## 8. Evolution of Seafood Barcoding through the Genomic Era

New genomic technologies (NGTs) have been developed over the last two decades. NGTs may lead to several and variegated advantages in the fields of food safety, agriculture, industry and pharmaceutics. A connection between genomics and food issues is also considered in “The Farm to Fork Strategy”, which is a focal point of the European Green Deal with the intent to make food systems fair, healthy and environmentally friendly. Among different approaches, DNA sequencing is still fundamental to trace elaborated foods that gather a mixture of sources within a single product. Although the traditional Sanger sequencing technology is still the gold standard for species identification, innovative approaches are emerging to analyse processed seafood samples that may contain more than one species [15]. According to the above-mentioned concepts, in the last decade, genomics techniques have become more common and exploited in all fields in science and have consequently led to technological improvements and a decrease in costs.

Alternative next-generation DNA barcodes have been proposed, starting from innovative approaches in taxonomy and population genetics [181]. In particular, Restriction Site Associated DNA (RAD) sequencing has been proposed to distinguish closely related species. This technique is very efficient at generating sequence data from many thousands of nuclear loci; however, taxon-specific optimization is requested and precludes the application of RAD sequencing as a universal barcoding approach. Approaches based on genome skim are mostly used for floristic species and will therefore not be considered in this manuscript, while a more common method is based on the use of capture probes. Probe sets are being developed for both mitochondrial and nuclear markers, and they may offer powerful extended barcodes. However, the need to develop probes having wide phylogenetic coverage is still a limiting step.

On the other hand, Next Generation Sequencing (NGS) has been proposed as the most valuable substitute to the classical Sanger approach. The major difference between Sanger technology and NGS is the capacity of the latter technique to identify up to 15 or more different fish species potentially present in a single highly processed product [182]. The progress of NGS has recently spread out as no other technique has done before. Its ability to sequence millions of small DNA fragments in parallel has revolutionized the genomic research. In fact, the simultaneous identification of animal and plant species in food products is one of the main goals to reach a reliable food safety using high-throughput sequencing formats [183,184].

Most of literature in this field has been increasing exponentially since the last 5 years, and the majority of approaches are nowadays managed through the Ion TorrentTM and IlluminaTM technologies. Kappel et al. [185] exploited metabarcoding using the Illumina MiSeq platform, targeting two short *cytb* fragments useful for tuna species identification in mixtures containing one to four species. Results provided precise sequence recoveries, allowing the identification of a minimum percentage of *Katsuwonus pelamis* (around 1% *w*/*w*) mixed inside major quantities of *Thunnus alalunga* within the same canned product. In the same year, Carvalho et al. [186] explored barcoding with the Ion torrent PGM method for the identification of fish species in highly processed codfish products, reporting a mislabelling rate of 41%. Giusti et al. [187] analysed surimi products, applying the same technology, using *16S* rDNA barcodes from a wide range of fish and cephalopod species. The authors verified that 37.5% of the products were mislabelled, 25% declared a species different from those identified and 25% did not label the presence of molluscs. Wang et al. [15] tested the feasibility of next-generation sequencing in identifying mixed salmon products sold in Shanghai. Salmon samples containing up to eight species were amplified using *16S* rDNA mini-barcode primers and sequenced on an Illumina HiSeq2500 platform. All species were accurately identified, and mixtures as low as 1% (*w*/*w*) could be detected. Both Sanger and NGS techniques were used to compare species identification, and a final cross-validation was obtained with real-time PCR to verify the accuracy of the DNA metabarcoding technology. DNA barcoding and metabarcoding of commercial salmon food products revealed the presence of mislabelling in 16 of 32 (50%) samples. The characterization of novel nuclear targets functional to flatfish samples identification was performed by Paracchini et al. [188] in silico study and NGS. Samples of various species of the Pleuronectidae family were analysed using short candidate nuclear regions. The advantages of these novel targets over the mitochondrial ones were demonstrated particularly for the capability to identify hybrid individuals, as well as multiple fish species in complex mixtures. More precisely, the ring finger protein 41 gene, also known as *Nrdp1*, showed the highest level of species differentiation, followed by the genes encoding the homeobox C13a/C13b proteins and midline 2 [188]. Two years later, the same research group [189] proposed additional nuclear genes and four non-coding regions assessed on raw or mildly treated commercial products. Using the NGS technique, gadoid species were successfully identified in complex mixtures and processed samples. 

Interestingly, the NGS method can recover complete or near-complete barcodes under challenging experimental conditions, even in century-old samples where DNA is highly degraded [190]. Nevertheless, NGS represents a valuable tool to detect bacterial contamination in seafood with the aim of avoiding food-borne diseases [191], which represent an additional threat to consumers in addition to species substitution, particularly in popular tourist destinations [58]. 

It must be remarked that the application of NGS in food science is quite a recent acquisition and therefore under continuous development. The constant search for the best approach in terms of time, costs, quality and efficiency to obtain entire mitogenome sequences is leading to new advances both in terms of laboratory activities and bioinformatics tools for data analysis. Continued improvements in sequencing platforms and analysis tools will make this approach even more reliable and cost effective very shortly [192].

## 9. Conclusions

To protect consumers from food frauds and health hazards and to improve the monitoring of species endangered by overfishing and illegal commercial activities, reliable molecular tools to perform barcoding DNA analysis were developed. In this review, we explored and presented a wide number of barcoding approaches with a special focus on fish species traceability both in terms of taxonomic identification and labelling. The proposed methods can identify with high accuracy the different species (or even lower taxa) in a wide range of raw and processed seafood products. For each method, a plethora of different studies has been discussed highlighting the main advantages and pitfalls.

Considering the overall evaluation and the highlighted differences between more classical markers and novel nuclear and mitochondrial barcoding regions, it is noteworthy that the choice for the best approach must still consider variegated aspects that have to be evaluated under an expert-based judgement. As a matter of fact, the combination of different methods to improve the accuracy scores of correct identifications seems the best practical way to reach a reliable species validation. Nevertheless, independent or integrated data-sharing platforms are nowadays available to align, validate and classify the generated barcoding sequences. 

The recent description and application of novel powerful genomics technologies open new perspectives. Innovative approaches based on NGS may generate millions of sequence reads in parallel, also under challenging experimental conditions, such as sample degradation due to bad preservation within the commercial pathway. Its power will have to be fully implemented in the future to reach constant improvements in terms of time, costs, quality and efficiency with the aim of increasing the application of DNA barcoding as one of most effective tools to discover unethical activities in seafood consumption. On the other hand, it must be remarked that constant technological improvements in the fields of molecular genetics and genomics make biotechnological approaches more reliable. Adaptation of protocols starting from DNA extraction coupled to enrichment techniques [192] up to bioinformatic elaboration of millions of available sequences [181] allows the presentation of barcoding as an affordable strategy becoming more and more popular, also considering the continuous fall of costs.

## Figures and Tables

**Table 1:** Comparison of advantages and disadvantages of mitochondrial and nuclear DNA.

	mtDNA	nDNA
	High number of copies of mtDNA	Useful when occur hybridization and introgression
Advantages	Matrilinear inheritance	
	Low variability within species and greater differentiation between	
Disadvantages	Nomenclature difficulties in differentiating closely related species	Subjected to recombination
		Single copy genes present in each cell

**Table 2:** Examples of seafood mislabelling concerning species included in the IUCN “Red List” detected with DNA barcoding.

Reference	Collected Sample	Species Discovered	Global “Red List”	World Region
	“Caçao” fish	*Carcharhinus brachyurus*	VU	Brazil
	“Caçao” fish	*Galeorhinus galeus*	CR	Brazil
	“Caçao” fish	*Gymnura altavela*	EN	Brazil
	“Caçao” fish	*Myliobatis goodei*	VU	Brazil
	“Caçao” fish	*Narcine brasiliensis*	NT	Brazil
	“Caçao” fish	*Prionace glauca*	NT	Brazil
	“Caçao” fish	*Pseudobatos horkelii*	CR	Brazil
Almeròn-Souza F. et al., 2018 [57]	“Caçao” fish	*Rhizoprionodon lalandii*	VU	Brazil
	“Caçao” fish	*Rhizoprionodon porosus*	VU	Brazil
	“Caçao” fish	*Sphyrna lewini*	CR	Brazil
	“Caçao” fish	*Sphyrna zygaena*	VU	Brazil
	“Caçao” fish	*Squalus mitsukurii*	EN	Brazil
	“Caçao” fish	*Squatina occulta*	CR	Brazil
	“Caçao” fish	*Xiphias gladius*	NT	Brazil
	“Caçao” fish	*Zapteryx brevirostris*	EN	Brazil
	*Engraulis encrasicolus*	*Thunnus albacares*	LC	Europe
	*Engraulis encrasicolus*	*Thunnus alalunga*	LC	Europe
	*Thunnus alalunga*	*Thunnus thynnus*	LC	Africa
Blanco-Fernandez C. et al., 2021 [54]	*Thunnus alalunga*	*Thunnus obesus*	VU	Africa
	*Merluccius capensis*	*Gadus morhua*	VU	Africa
	*Katsowomus pelamis*	*Thunnus thynnus*	LC	Italy
Filonzi L. et al., 2021 [32]	*Katsowomus pelamis*	*Thunnus maccoyii*	EN	Italy
	*Lamna nasus*	*Isurus oxyrinchus*	EN	Italy
Lowenstein J.H. et al., 2009 [46]	*Thunnus* sp.	*Thunnus thynnus*	LC	USA
	*Thunnus* sp.	*Thunnus maccoyii*	EN	USA
Dufflocq P. et al., 2022 [55]	*Xiphias gladius*	*Isurus oxyrinchus*	EN	Chile
	*Xiphias gladius*	*Lamna nasus*	VU	Chile
Ferrito V. et al., 2019 [51]	*Xiphias gladius*	*Mustelus mustelus*	EN	Italy
	*Xiphias gladius*	*Oxyntus centrina*	EN	Italy
	*Conger* sp.	*Micropogonias furnieri*	LC	Brazil
	*Salmo salar*	*Thunnus alalunga*	NT	Brazil
	*Thunnus* sp.	*Seriola zonata*	LC	Brazil
	*Thunnus* sp.	*Lepidocybium flavobrunneum*	LC	Brazil
	*Thunnus* sp.	*Salmo salar*	LC	Brazil
	*Thunnus* sp.	*Seriola lalandii*	LC	Brazil
	“White fish”	*Thunnus obesus*	VU	Brazil
	“White fish”	*Salmo salar*	LC	Brazil
Staffen C.F. et al., 2017 [58]	*Peprilus paru*	*Micropogonias furnieri*	LC	Brazil
	*Micropogonias undulatus*	*Prionace glauca*	NT	Brazil
	*Lepidocybium flavobrunneum*	*Ruvettus pretiosus*	LC	Brazil
	Flounder gen.	*Isopisthus parvipinnis*	LC	Brazil
	Flounder gen.	*Micropogonias furnieri*	LC	Brazil
	*Epinephelus* sp.	*Micropogonias furnieri*	LC	Brazil
	*Molva* sp.	*Micropogonias furnieri*	LC	Brazil
	*Pangasius pangasius*	*Micropogonias furnieri*	LC	Brazil
	*Salmo salar*	*Seriola zonata*	LC	Brazil
	*Salmo salar*	*Prionace glauca*	NT	Brazil
	*Salmo salar*	*Micropogonias furnieri*	LC	Brazil
	*Carcharias taurus*	*Prionace glauca*	NT	Brazil
	*Xiphias gladius*	*Trichiurus lepturus*	LC	Brazil
	*Cynoscion regalis*	*Isopisthus parvipinnis*	LC	Brazil
Barbuto M. et al., 2010 [59]	*Mustelus mustelus*	*Squalus acanthias*	VU	Italy
	*Mustelus mustelus*	*Prionace glauca*	NT	Italy
	*Mustelus mustelus*	*Galeorhinus galeus*	CR	Italy
	*Mustelus mustelus*	*Alopias superciliosus*	VU	Italy
	*Mustelus mustelus*	*Isurus oxyrinchus*	EN	Italy

**Table 3:** DNA barcoding principal database specifications.

Database	Molecular Markers	Taxa	N° of Sequences	World Region
GenBank	All	All	239 milions	All
BOLD	COI, ITS, rbcL, matK	Animal, Plants, Fungi, Protists	11 milions	All
FISH-BOL	COI	Freshwater and Marine Species	Not Available	All
Fish-Trace	cytb, rhodopsin	Marine Species	Not Available	Europe
Aquagene	COI, cytb, MYH6, rhodopsin	Marine Species	1383	Central Eastern Atlantic

## Data Availability

Data is contained within the article.
